# Dr. Jacob Moyer Hagey

**Published:** 1909-09

**Authors:** 


Dr. Jacob Moyer Hagey, died at his home at Mount Morris, N. Y., August 3, 1909, aged 76 years. He was ill for several months, caused by successive strokes of paralysis. For some time he had been in a critical condition, being unable to talk or help himself in any way. He graduated from Rush Medical College, Chicago, in 1862.

He was a member of many societies, among which were, Livingston County Historical, Central New York Medical Association, American Medical Association and the American Public Health Association.. He is survived by his wife, two daughters, Misses Maude and Blanche Hagey, and one son. Dr. John B. Hagey, of Illinois.

